# Construction of Coexpression Networks Affecting Litter Size in Goats Based on Transcriptome Analysis

**DOI:** 10.3390/ani15111505

**Published:** 2025-05-22

**Authors:** Yifan Ren, Junmin He, Guifen Liu, Chen Wei, Xue Li, Jingyi Mao, Guoping Zhang, Wenhao Zhang, Li Long, Ming Wang, Kechuan Tian, Xixia Huang

**Affiliations:** 1 College of Animal Science, Xinjiang Agricultural University, Urumqi 830052, China; 15136225372@163.com (Y.R.); lixueli1126@163.com (X.L.); zwh22635@163.com (W.Z.); ll2057138137@gmail.com (L.L.); xjming2024@163.com (M.W.); 2 Institute of Animal Science and Veterinary Medicine, Shandong Academy of Agricultural Sciences, Jinan 250100, China; hejunmin330@163.com (J.H.); liuguifen126@126.com (G.L.); weichenchen1989@126.com (C.W.); m2797611850@163.com (J.M.); gpzhangsaas@163.com (G.Z.)

**Keywords:** goat, litter size, transcriptome sequencing, WGCNA

## Abstract

Optimal litter size on goat farms is an important trait for production and economic efficiency. The ovary and uterus, key components of the reproductive system, play essential roles in reproductive performance. In recent years, numerous genes linked to goat reproductive performance have been identified. However, reliable marker genes that are specifically associated with litter size require further exploration. In this study, eight Jining Grey goats were divided into high-yield (*n* = 4) and low-yield (*n* = 4) groups on the basis of their kidding records to identify key regulatory genes associated with litter size. Ovarian and uterine tissues were collected during oestrus for RNA sequencing (RNA-seq). After two outlier uterine tissue samples were excluded, the remaining 14 samples were subjected to WGCNA and differential expression gene (DEG) analysis. A total of 1224 DEGs were identified (|log2(fold change) ≥ 1|, *p* ≤ 0.05), including 912 in ovarian tissues (monozygotic vs. polyzygotic, MO vs. PO) and 312 in uterine tissues (MU vs. PU). Through WGCNA, we identified 15 coexpression modules, among which four key modules were significantly correlated with litter size. Our analysis focused on the magenta and green modules, as they contained 11 and 3 candidate genes overlapping with the DEGs, respectively. Notably, three genes—*FOXC1*, *FOSB*, and *FGL2*—were found to play important roles in both ovarian and uterine tissues. These genes mainly participate in regulatory processes such as RNA polymerase II transcription factor activity, calcium ion binding, and extracellular space organization, highlighting their potential as key candidates for future research. Overall, we identified several gene modules associated with litter size in goats, providing potential molecular markers for investigating litter size traits in Jining Grey goats.

## Simple Summary

Litter size is a critical trait affecting the reproductive performance of goats. Understanding its molecular mechanisms can provide genetic insights into multiple kidding in goats. In this study, we analysed transcriptomic data from the ovarian and uterine tissues of Jining Grey goats during oestrus. Using weighted gene coexpression network analysis (WGCNA), we constructed a coexpression network related to litter size and identified key modules and genes associated with reproduction. These findings provide a theoretical foundation for molecular breeding aimed at improving the reproductive performance of goats.

## 1. Introduction

Goats (*Capra hircus*) are important sources of high-quality protein and raw materials for humans. With rapid economic development and changing consumer preferences in China, the demand for goat meat has steadily increased, driving higher production of livestock products. As the world’s largest producer of goat meat, China has also ranked among the top three importers over the past decade, indicating a significant gap between supply and demand [1]. Most local goat breeds in China are single-kidding, making high fertility a desirable and genetically relevant trait for goat meat production. Even slight improvements in litter size can significantly benefit farmers [2]. Among the many factors that influence goat reproductive performance, litter size has particular importance; however, the molecular mechanisms underlying this trait remain limited. Since the discovery of the impact of the *FecB* gene on sheep fertility [3], several genes associated with litter size in sheep have been identified, including *GDF9*, *BMP15*, *VAV3*, *GABRG1*, *FNDC1*, *LEPR*, and *CCDC63* [4,5,6]. Although no universally accepted candidate genes for litter size have been identified in goats, recent studies have reported several promising associations. For example, *AMH* was linked to prolificacy in Chuan Zhong black goats [7], while SNPs in *INHA* and *ACVR2B* were associated with litter size in Dazu black goats [8]. In Jining Grey goats, RPL4 [9] and the *GLUD1* [10] allele A were proposed as potential markers for improving reproductive performance. These findings highlight the growing understanding of genetic contributors to litter size in goats, although most studies have focused on individual genes or specific breeds. Therefore, identifying key genes associated with litter size in goats will enhance our understanding of the genetic basis of reproduction and provide valuable molecular markers for optimizing genetic breeding programs.

The ovary is the main site of follicular development and plays an important role in regulating the secretion of reproductive hormones, which is the basic condition for guaranteeing reproductive performance. In goats, recent transcriptomic studies have begun to reveal gene expression differences between high- and low-fertility individuals [11]. In sheep, more extensive research has been conducted using approaches such as noncoding RNA profiling and WGCNA to identify candidate genes and regulatory networks [12,13,14]. The number of kids produced is influenced by several factors, such as the oocyte fertilization rate, embryo survival and uterine receptivity [15]. The uterus is essential for embryo implantation and pregnancy maintenance. It also significantly impacts kidding performance and reproductive efficiency in goats [16]. Although the uterus has an important role in litter size, research on the mechanisms by which the uterus affects litter size in goats is still limited [17]. Therefore, a combined analysis of ovarian and uterine transcriptomes may offer novel insights into the regulatory mechanisms underlying litter size in goats.

In recent years, with improvements in living standards, the demand for high-quality goat meat has gradually increased [18]. The high costs of traditional breeding methods have limited the rapid improvements of goat fertility. WGCNA, an effective approach based on coexpression module construction [19], has been widely used to identify key regulatory modules and genes for important traits in goats, such as meat quality [20], muscle development [21], and reproductive performance [22]. By combining differential expression analysis with WGCNA, this approach enables a more powerful identification of candidate genes associated with complex traits such as litter size.

The Jining Grey goat is a famous local breed with excellent characteristics, such as high fertility and early sexual maturity, and is the best animal model for studying candidate genes affecting the number of kids produced in goats. In this study, based on transcriptome sequencing data from the ovarian and uterine tissues of Jining Grey goats with different litter sizes, key expression modules and candidate genes affecting the litter sizes of goats were identified by WGCNA. The results of this study will help elucidate the mechanism by which the ovary and uterus affect litter size and provide an important theoretical basis for the breeding of highly propagated lines of local goat breeds.

## 2. Materials and Methods

### 2.1. Test Animals and Sample Collection

This study was approved by the Experimental Animal Ethics Committee of the Institute of Animal Husbandry and Veterinary Medicine, Shandong Academy of Agricultural Sciences (IASVM-2022-003). The experimental animals were Jining Grey does bred at the National Jining Grey Goats Breeding Farm in Jiaxiang County, Jining, China. Based on the kidding number records of 193 Jining Grey does, 8 non-pregnant does aged 3–4 years were selected. They were divided into two groups: a monotocous group (with 3 consecutive litters of single kids) and a polytocous group (with no fewer than 3 kids per litter for 3 consecutive litters), with 4 samples in each group. The selected goats were not genetically related. In accordance with the National Standard Specifications for Performance Measurement of Sheep and Goats (NY/T 1236-2006) [23], the body weight, height, body length, chest girth, and cannon bone circumference of the experimental does were measured (Table 1). All does were maintained under standardized housing and feeding conditions and were healthy and free from disease. Oestrus synchronization was achieved by inserting a CIDR (Zoetis Inc., Parsippany, NJ, USA) into the vagina. On day 14, the CIDR was removed, and 300 IU of pregnant mare serum gonadotropin (PMSG, Ningbo Second Hormone Factory, Ningbo, China) was injected. Bucks were used for teasing three times per day, and the experimental does were considered to be in oestrus when they allowed mounting and showed visible signs of oestrus, such as vulvar swelling and redness. Tissue samples were collected within 12 h of oestrus onset. To minimize physiological variability at the time of sampling, the oestrous cycles of all experimental does were synchronized. Ovarian (O) and uterine (U) tissues were collected from monotocous (M) and polytocous (P) does and designated as MO, PO, MU, and PU, respectively (Table 1). Based on morphological assessment during collection, the ovaries from both groups appeared to be at comparable physiological stages. All tissues were rinsed with PBS(Biosharp Life Sciences, Hefei, China) buffer, cut into small pieces, placed into 2 mL cryotubes, labelled, flash-frozen in liquid nitrogen, and stored at −80 °C until further use. The left ovary and a standardized portion of uterine tissue were used for total RNA extraction.

### 2.2. RNA Extraction and Library Construction

Total RNA was extracted from the ovarian and uterine tissues of eight Jining Grey goats using the TRIzol^®^ reagent (Invitrogen, San Diego, CA, USA). Tissue samples (50–100 mg) were collected, and total RNA was extracted following the manufacturer’s instructions. RNA concentration and quality were assessed using a NanoDrop One spectrophotometer (Thermo Fisher Scientific, Wilmington, DE, USA), ensuring that the A260/A280 values ranged between 1.8 and 2.1 and the A260/A230 values were greater than 2.0. RNA integrity was checked by 1.5% agarose gel electrophoresis. Transcriptome library construction was performed by BGI Genome Co., Ltd. (Shenzhen, China) (https://biosys.bgi.com, accessed on 21 March 2025), and PE 100 bp sequencing was conducted using the DNBSEQ sequencing platform (BGI Genomics, Shenzhen, China).

Prior to the analysis, the raw data were filtered using SOAPnuke v2.3 (removing adaptors, rRNA, and low-quality reads) [24] and checked for GC content to obtain clean reads. The goat reference genome ARS1 (https://www.ncbi.nlm.nih.gov/datasets/genome/GCF_001704415.1, accessed on 21 March 2025) was indexed by HISAT2 (v2.0.4) [25] and aligned using Bowtie2 (v2.2.5) [26] to obtain the mapping information of the reads in the reference genome. RSEM (v1.2.12) [27] was used to determine the expression levels of genes and transcripts, and the fragments per kilobase per million (FPKM) values were calculated [28]. FPKM values were used solely for downstream analyses, including WGCNA construction and sample clustering, whereas DESeq2 was applied to the raw count data for DEG identification.

### 2.3. Differential Expression and Enrichment Analysis

Count values of genes in the ovarian and uterine tissue samples were analysed for differential expression using DESeq2 (v1.44.0) package [29], with screening criteria |log2(fold change) ≥ 1| and *p* ≤ 0.05. In this study, the low-litter size group (M) was used as the reference group, and the high-litter size group (P) was used as the treatment group. Thus, a positive log2 fold change indicates upregulation in the P group compared to the M group. Gene Ontology (GO) and Kyoto Encyclopedia of Genes and Genomes (KEGG) pathway analyses were performed using the DAVID database (https://david.ncifcrf.gov/, accessed on 21 March 2025) [30], and the selected genes were imported into the OmicShare online platform for visualisation (https://www.omicshare.com/tools, accessed on 21 March 2025). Considering that Gene Set Enrichment Analysis (GSEA, https://www.gsea-msigdb.org/gsea/index.jsp, accessed on 21 March 2025) [31] does not require a fixed cut-off for differential gene expression and has a broader functional scope, GSEA–GO enrichment analysis was performed on the entire gene sets of the ovary and uterus, with a significance threshold of *p* < 0.05.

### 2.4. Weighted Gene Coexpression Network Analysis (WGCNA)

Among the eight Jining Grey goats, genes with FPKM < 1 across all samples were excluded, and a coexpression network was constructed for the remaining genes. The WGCNA package (v1.7.1) [19] in R (v4.1.2; https://www.r-project.org, accessed on 21 March 2025) was used to construct a weighted gene coexpression network based on the filtered FPKM values and litter size phenotypic data. The eight samples were first analysed by hierarchical clustering analysis was performed using the hclust function, and samples with abnormal expression patterns were excluded at a threshold of 15,000. The Pearson correlation coefficients between gene pairs were calculated based on the screened samples using the pickSoftThreshold (sft) function to construct the neighbour-joining matrix. The scale-free topology index (R^2^) was set to 0.85 as the optimal soft-threshold value (β). The adjacency matrix was converted to a topological overlap matrix (TOM) using the adjacency function, forming a weighted gene coexpression network. Genes with similar expression patterns were clustered into modules and assigned colours. After module construction, the module eigenvalue (ME) was calculated for each module. The Pearson correlation coefficient (r) and significance (*p*-value) between each ME and litter size were determined, and the module with the highest absolute correlation (|r|) and lowest *p*-value was selected as the target module. The genes in the target module were subsequently analysed for GO and KEGG enrichment analysis using the DAVID database (https://david.ncifcrf.gov/, accessed on 21 March 2025). GO terms and pathways with significant gene enrichment (*p* < 0.05) were identified [32].

### 2.5. Identification of Pivotal Genes Associated with Ovarian and Uterine Litter Sizes

To further screen the pivotal genes regulating litter size from the target modules, the genes identified by WGCNA were further screened. First, the correlation between gene expression and module eigengene (ME) and gene expression (GS, correlation with litter size phenotype) were calculated. Genes in the target module were selected based on the criteria of |MM| > 0.85 and |GS| > 0.35. The screened genes were then imported into the STRING database (https://cn.string-db.org, accessed on 21 March 2025) [33], and a protein–protein interaction (PPI) network was constructed. The PPI network was analysed using the cytoNCA plug-in [34] in Cytoscape (v3.9.1; https://cytoscape.org, accessed on 21 March 2025) [35]. Degree values were determined, and the top 30 nodes were selected as candidate genes for key modules and visualised. The candidate genes were then compared with two sets of differentially expressed genes (DEGs) to identify the final hub gene of the key module.

## 3. Results

### 3.1. Differential Gene Screening

**Figure 1:**
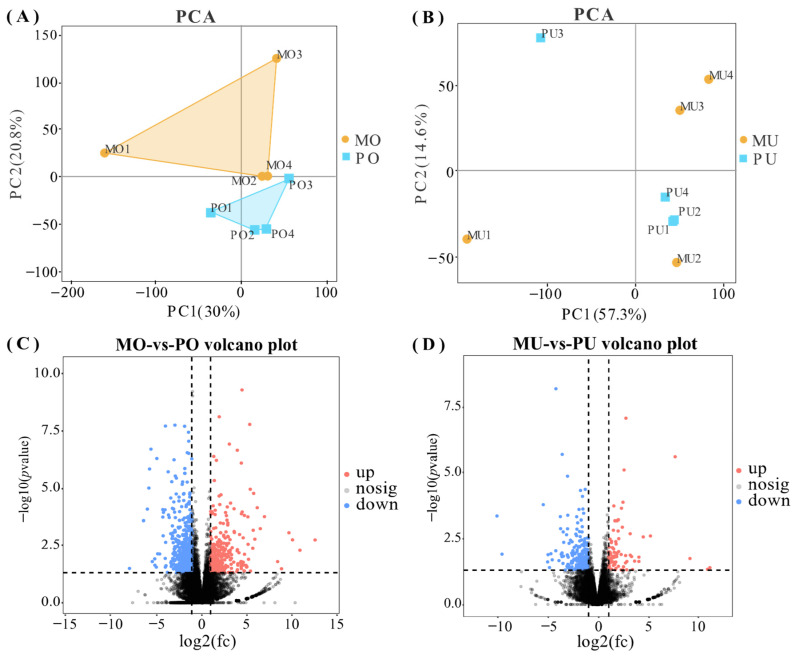
Representation of mRNA expression levels. (**A**) PCA plot of ovarian tissue. (**B**) PCA plot of uterine tissue. (**C**) Volcano plots of differentially expressed genes in the ovarian tissues of the high- and low-yield groups. (**D**) Volcano plots of differentially expressed genes in the uterine tissues of the high- and low-yield groups. Red indicates upregulation and blue indicates downregulation.

Principal component analysis (PCA) was performed on the samples to compare the gene expression profiles between the two groups in the ovarian and uterine tissues separately. In ovarian tissue, the global gene expression profiles of the two groups were clearly segregated, indicating distinct transcriptional differences (Figure 1A). In contrast, in uterine tissue, two significant outlier samples (MU1 and PU3) were identified and excluded from further analysis to ensure data reliability (Figure 1B). Differential expression analysis revealed a total of 912 DEGs in ovarian tissue, including 337 upregulated and 575 downregulated genes (Figure 1C). In uterine tissue, 312 DEGs were identified, with 82 upregulated and 230 downregulated genes (Figure 1D).

### 3.2. Functional Enrichment Analysis of DEGs

#### 3.2.1. GO Enrichment Analysis

**Figure 2:**
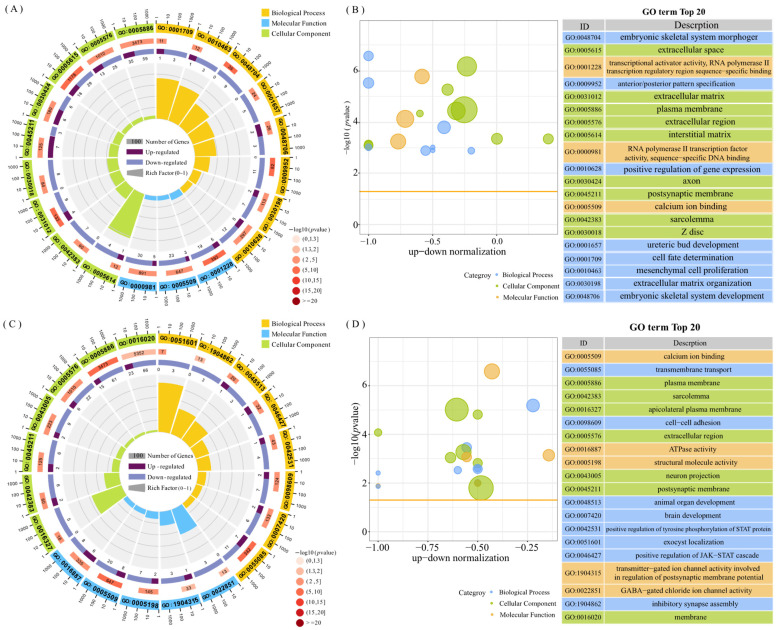
Top 20 entries for the GO enrichment analysis of DEGs. (**A**) Circle plot of GO-enriched entries for MO vs. PO DEGs. (**B**) Bubble plot of GO-enriched entries for MO vs. PO DEGs. (**C**) Circle plot of GO-enriched entries for MU vs. PU DEGs. (**D**) Bubble plot of GO-enriched entries for MU vs. PU DEGs (circle plot: from the outside to the inside, the first circle represents the first 20 enriched pathways, and the numbers outside the circle are the gene numbers of the pathway; the second circle represents the number of background genes in the pathway—the more genes there are, the longer the bands are; the third circle represents the number of DEGs in the pathway, darker colours are upregulated differential genes, and lighter colours are downregulated differential genes; and the fourth circle represents the values of the Rich Factors in each pathway).

The GO enrichment analysis revealed that 105 GO terms were significantly enriched in ovarian tissues and 45 GO terms in uterus tissues. In ovarian tissues, the DEGs were primarily enriched in biological processes such as embryonic skeletal system, molecular functions including calcium binding, and cellular components such as extracellular region (Figure 2A,B). In uterine tissues, the DEGs were enriched for terms related to intercellular adhesion, animal organ development, brain development, and GABA-gated chloride channel activity (Figure 2C,D). Among the enriched GO terms, 11 were shared between the two tissues, including extracellular space, transcriptional activator activity, sequence-specific binding of the RNA polymerase II transcriptional regulatory region, calcium binding, and multicellular biological development.

#### 3.2.2. KEGG Enrichment Analysis

**Figure 3:**
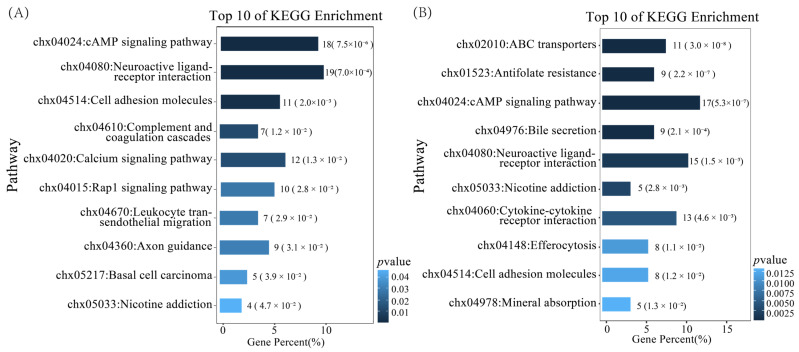
Top 10 pathways for KEGG enrichment analysis of DEGs. (**A**) KEGG-enriched pathways of the MO vs. PO differential genes. (**B**) KEGG-enriched pathways of the MU vs. PU differential genes.

KEGG enrichment analysis identified 11 enriched pathways in the MO vs. PO group and 16 in the MU vs. PU group. In the MO vs. PO group, the significantly enriched pathways included the cAMP signalling pathway, calcium signalling pathway, and cell adhesion molecules, among others (Figure 3A). In the MU vs. PU group, significantly enriched signalling pathways included antioxidant pathways, neuroactive ligand–receptor interaction, and cell adhesion molecules, along with other pathways (Figure 3B). Notably, both MO vs. PO and MU vs. PU groups shared five enriched signalling pathways, including the cAMP signalling pathway, neuroactive ligand–receptor interactions, and cell adhesion molecules. These results suggest the potential existence of key regulatory genes associated with litter size that are shared between ovarian and uterine tissues.

#### 3.2.3. GSEA Enrichment Analysis

**Figure 4:**
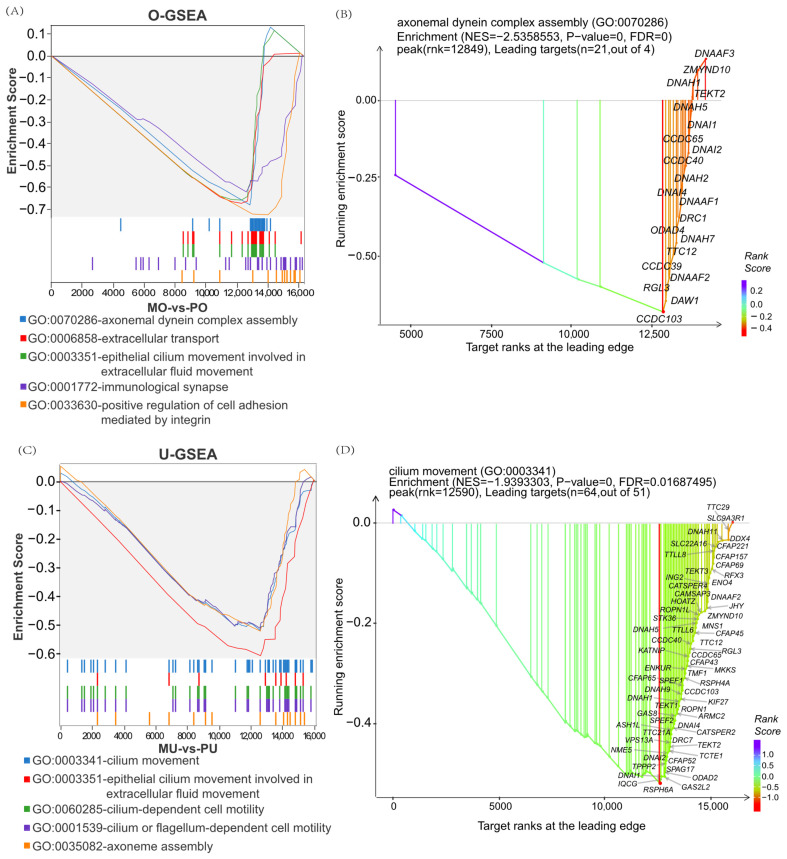
GSEA enrichment results of DEGs in the ovary and uterus. (**A**) GSEA enrichment results of MO vs. PO DEGs. (**B**) xGSEA dot plot of axonal dynamin complex entry gene sets. (**C**) GSEA enrichment results of MU vs. PU DEGs. (**D**) xGSEA dot plot of the gene set of the steroid signalling pathway.

To further explore the genetic mechanisms underlying the differences between the high- and low-yield groups, GSEA was performed. The results showed that, in the MOs vs. PO group, the enriched terms mainly included the axonal dynamin complex, extracellular transport, and related processes (Figure 4A). Among these, *DRC1*, *DNAI4*, *ODAD2*, and *CCDC40* played key regulatory roles in the ovarian tissues (Figure 4B). Interestingly, in the MU vs. PU group, the enriched pathways were predominantly associated with ciliary motility, epithelial cilia motility involved in extracellular fluid motility, and related processes (Figure 4C). Notably, *CFAP70*, *DNHD1*, *TPPP2*, and *CFAP251* genes were found to play important roles in the uterine tissues of goats (Figure 4D). In addition, epithelial cilia motility in reproduction-related pathways involved in extracellular fluid movement was present in both the ovary and uterus, ranking among the top five enriched terms in the GSEA analysis (Figure 4A,C), suggesting that cilia-related processes may play a pivotal role in regulating reproductive performance in goats.

### 3.3. Weighted Gene Coexpression Network Construction

**Figure 5:**
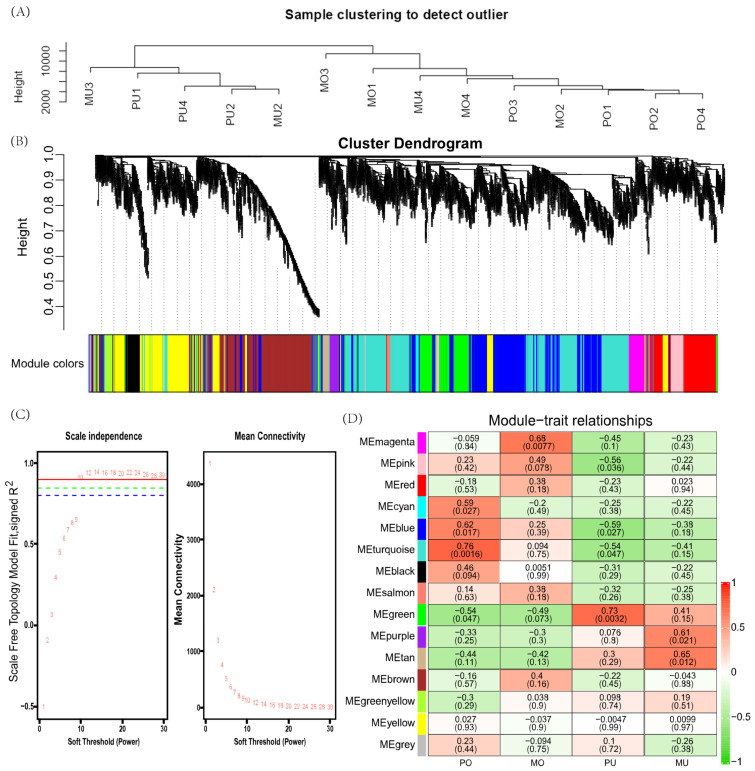
Weighted gene coexpression network analysis. (**A**) Module clustering map; (**B**) gene dynamic shear clustering tree, with each colour representing a module; (**C**) soft threshold determination of gene coexpression networks; (**D**) module–trait relationships, with each row representing a module–trait gene sum. Each cell contains the corresponding correlation and *p* value in parentheses. Red indicates positive correlations, green indicates negative correlations, and the darker the colour is, the stronger the correlation. Each set of data represents the correlation coefficient r value of the module with the phenotype, and the values in parentheses are the significant *p*-values.

After preprocessing the data, 13,081 genes were obtained for WGCNA analysis. The results showed that the 14 samples clustered well (Figure 5A). The optimal soft threshold was determined to be 10, with a scale-free topology fit index (R^2^) of 0.9 (Figure 5C). Using the dynamic shear tree model with the minimum module size of 35 genes, 15 coexpression modules were obtained (Figure 5B). Based on the correlation (r) and *p*-value between module eigengenes and kidding traits, the key modules most relevant to the four traits were identified for further analysis. The turquoise module was significantly and positively correlated with PO (r = 0.73, *p* = 0.041) and contained 3044 genes. The magenta module was significantly and negatively correlated with MO (r = −0.74, *p* = 0.035) and contained 363 genes. The green module was significantly and positively correlated with PU (r = 0.73, *p* = 0.0032) and contained 1021 genes. The tan module was significantly and positively correlated with the MU (r = 0.65, *p* = 0.012) and contained 140 genes (Figure 5D).

**Figure 6:**
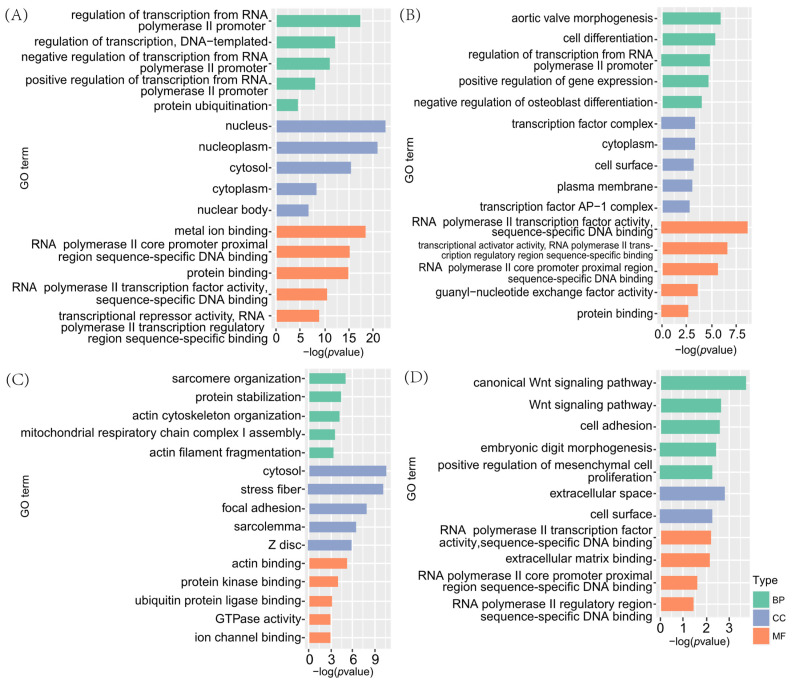
GO annotations of genes within key modules associated with multiple births. (**A**) Turquoise module, (**B**) magenta module, (**C**) green module, and (**D**) tan module.

Genes within the four key modules were analysed for GO enrichment. The results (Figure 6) showed that genes in the turquoise module were significantly enriched in 235 GO terms, primarily related to transcription regulation by the RNA polymerase II promoter, cytoplasmic lysis, metal ion binding, and protein binding. In the magenta module, 96 significantly enriched GO terms were associated mainly with cell differentiation and transcription factor regulation, including positive regulation of gene expression, negative regulation of osteoblast differentiation, and transcription factor complexes. Genes in the green module were significantly enriched in 113 GO terms, most of which were actin-related, such as myosin nodule organisation, actin cytoskeleton organisation, stress fibres formation, and actin binding. Although the tan module contained only 24 significantly enriched GO terms, it included pathways associated with embryonic development, such as the classical Wnt signalling pathway, cell adhesion, embryonic finger morphogenesis, and extracellular space.

**Figure 7:**
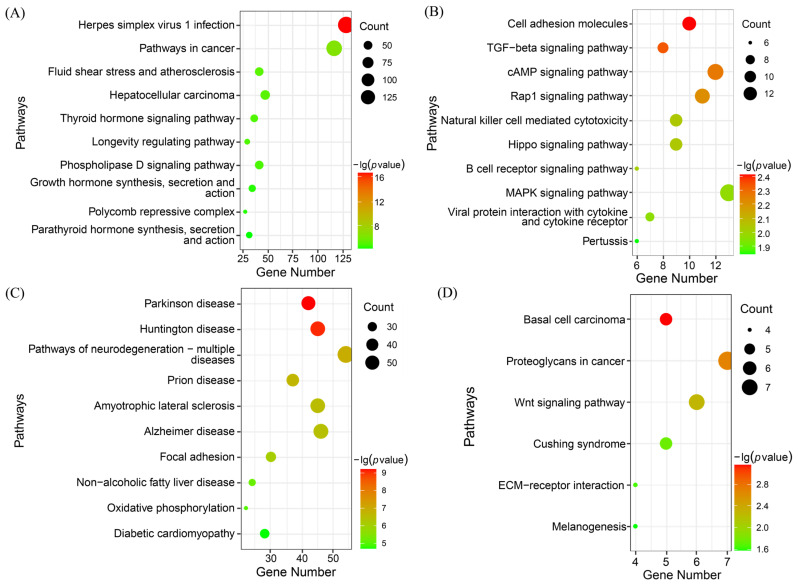
KEGG pathways of key genes associated with multiple rounds of fecundity. (**A**) Turquoise module, (**B**) magenta module, (**C**) green module, and (**D**) tan module.

The KEGG enrichment analysis showed that all four key modules were significantly enriched (Figure 7). Genes in the turquoise module were significantly enriched in 76 pathways, including the Wnt, MAPK, GnRH, and AMPK signalling pathways. The magenta module was significantly enriched in 17 pathways, including cell adhesion molecules, the cAMP signalling pathway, and the Hippo signalling pathway. Genes in the green module were significantly enriched in 45 pathways, including the pathways of neurodegeneration—multiple diseases, the calcium signalling pathway, the PI3K-Akt signalling pathway, iron death, and 45 additional pathways. The tan module was significantly enriched in six signalling pathways, including the basal cell carcinoma pathway.

### 3.4. Hub Gene Identification

**Figure 8:**
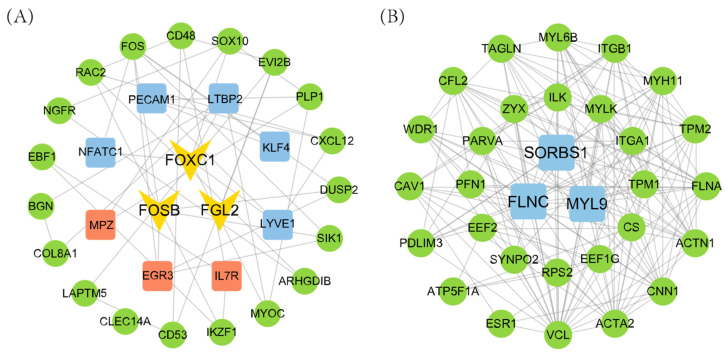
PPI network interactions of target module genes. (**A**) Magenta module; (**B**) green module. The blue boxes represent ovarian hub genes, the red boxes represent uterine hub genes, and the V shapes represent ovarian and uterine hub genes.

To further identify key regulatory genes in the key module, a protein–protein interaction (PPI) network was constructed (Figure 8). The candidate genes from the key modules were compared with the DEGs in the ovary and uterus tissues to obtain the functionally relevant genes in each module. Analysis revealed 11 high-pivotal genes in the magenta module and 3 in the green module. The remaining 2 modules did not contain genes overlapping with DEGs. Therefore, only the magenta and green modules were retained for downstream hub gene identification and discussion. A total of 11 genes (*FOXC1*, *FOSB*, *FGL2*, *FLNC*, *SORBS1*, *MYL9*, *KLF4*, *LTBP2*, *PECAM1*, *NFATC1*, and *LYVE1*) were ultimately identified as influencing ovarian reproduction traits, while 6 pivotal genes (*FOSB*, *FGL2*, *FOXC1*, *EGR3*, and *IL7R*) were associated with litter size regulation through uterine function. Notably, *FOXC1*, *FOSB*, and *FGL2* were differentially expressed in both the ovary and uterus. These genes were also among the top 30 in terms of connectivity within the PPI network, suggesting their pivotal role in regulating litter size through both ovarian and uterine mechanisms.

## 4. Discussion

Litter size is a key economic trait in goats that directly influences reproductive performance. Understanding its molecular regulatory mechanisms is essential for improving breeding efficiency. The ovary and uterus, as critical reproductive organs, are closely associated with litter size. Previous studies on fertility have primarily focused on ovulation rate and follicular development in the ovary, with less emphasis on uterus function [36,37,38]. However, as the site of foetal development, the uterus plays a crucial role in litter size, with factors such as uterine tolerance and embryo implantation success significantly influencing the number of kids delivered [39,40,41]. Here, we systematically analysed the ovarian and uterine transcriptomes of Jining Grey goats with different litter sizes. By integrating differential expression analysis and weighted gene coexpression network analysis (WGCNA), we identified key regulatory pathways and hub genes associated with prolificacy. These findings provide molecular insights into the mechanisms underlying litter size variation in goats.

To investigate the biological functions of these DEGs in the ovaries and uterus, differential gene enrichment analyses were performed. GO and KEGG enrichment analyses revealed that DEGs in the two tissues were enriched in 11 GO terms and 5 KEGG pathways, several of which have been shown to play regulatory roles in fecundity, including transcriptional regulation of RNA polymerase II [42], calcium ion binding [43] neuroactive ligand–receptor interactions [44,45], and the cAMP signalling pathway [46]. The remaining co-enriched terms may also be involved in reproduction regulation, such as extracellular space, plasma membrane, cell adhesion molecules, nicotine addiction, and cytotoxicity. This result is consistent with the findings by Zou et al., who studied ovarian gene expression during oestrus in Chuan Zhong Black goats with different litter sizes [47]. Similar enrichment results were obtained in a study of key pituitary genes related to litter size in sheep [5].

GSEA analysis was able to complementthe findings from differential gene enrichment analyses. GSEA–GO analysis revealed that both the ovary and uterus were significantly enriched for epithelial cilia motility involved in extracellular fluid movement, a result consistent with single-nucleus transcriptomics data on the molecular mechanism of litter size in goat ovarian cells [36]. Mammalian spermatozoa require uterine capacitation and structural modifications for fertilization [48,49]. According to the results of the present study, most of the significantly enriched genes in uterine tissue identified by GSEA–GO were associated with ciliary motility and sperm viability, suggesting that their involvement in sperm function. These findings indicate that differentially expressed ovarian and uterine genes play direct or indirect roles in fertility regulation.

WGCNA is an effective biological analysis method that integrates gene expression data with phenotypic information to identify key regulatory genes. In this study, correlations between phenotypes and gene modules were calculated based on the WGCNA results, and four key modules were initially identified: the turquoise, magenta, green, and tan modules. A protein–protein interaction (PPI) network was then constructed for each module, and their top 30 hub genes were intersected with differentially expressed genes (DEGs). Overlaps were found only in the magenta and green modules, suggesting their stronger biological relevance to litter size regulation. Therefore, the discussion primarily focuses on these two modules. The genes in these key modules were screened and intersected with the DEGs to ultimately identify the hub genes affecting both the ovaries and uterus. The magenta and green modules shared six significantly enriched GO terms, namely transcriptional activator activity, sequence-specific binding of RNA polymerase II transcriptional regulatory regions, calcium binding, cell surface, plasma membrane, extracellular space, and multicellular biogenesis. However, no overlapping pathways were identified in the KEGG enrichment analysis. Notably, DEGs from both tissues were also co-enriched in these six GO terms, which suggests that the genes enriched in these GO categories play important roles in litter size regulation in goats. Furthermore, the similarity in findings between differential gene analysis and WGCNA highlights the robustness and reliability of the methodology and results in this study. Comparable methodologies have been applied in goat reproductive studies. For instance, Zhang et al. identified *RPL4* as a candidate gene associated with litter size in ovarian tissue [9], while Sun et al. uncovered candidate molecular markers related to reproduction in oestrous goats through integrated proteomics and transcriptomics of the oviduct [22]. These findings further underscore the utility of combining WGCNA with differential analysis in identifying key reproductive genes.

RNA polymerase II, a 12-subunit enzyme with a highly complex structure, transcribes all coding genes as well as most noncoding RNAs in eukaryotic genomes [50]. Many studies have demonstrated its role in meiosis in mammals [51,52], early embryogenesis [53,54,55], and other reproductive processes. Calcium ions, as the most prevalent second messengers, regulate synapse formation, gene transcription, and the secretion of various hormones [56]. Studies in pigs [57,58], goats [59], sheep [5], and poultry [60] have shown that calcium ion binding significantly influences reproductive performance. In addition, calcium ions play a critical role in regulating hormone secretion in the hypothalamic–pituitary–gonadal (HPG) axis, mainly through concentration-dependent mechanisms [61,62,63].

The cell surface is a complex system composed of the plasma membrane, associated proteins, and cytoskeleton [64]. Vesicles, which are closely related to the extracellular cellular space, play essential roles in cellular transport and communication. During cytokinesis, vesicles fuse with the plasma membrane, delivering substances to the cell surface or release them into the extracellular space [65]. Qin et al. conducted comprehensive proteomic and transcriptomic analyses of goat ovaries, revealing that their differential gene enrichment results were consistent with those of the present study at the cellular component level [59]. All three important cellular component entries were located in the extracellular region, suggesting that the gene products identified in this study are secreted to play important roles outside the cell. The development of multicellular organisms is an extremely complex process involving the orderly regulation of cellular proliferation and differentiation to form an functional organism [66]. However, studies on this process in goat reproduction remain limited, whereas more extensive research has been conducted in species such as sheep [45] and monkeys [67].

Based on differential gene, WGCNA, and PPI analyses, three key ovarian and uterine genes affecting litter size in goats were identified: *FOXC1*, *FOSB*, and *FGL2*. *FOXC1* is a transcription factor encoding a protein comprising 553 amino acids [68]. It has been reported to play important roles in the development of the eye [69], kidney [70], and cardiovascular system [71]. In addition, *FOXC1* is involved in female fertility regulation and is essential for early ovarian development [72] and follicular maturation [73]. An et al. studied follicular development-related genes in goats and reported that upregulation of *FOXC1* expression promotes apoptosis in ovarian granulosa cells [74]. In this study, *FOXC1* and *FOSB* were primarily enriched in RNA polymerase II-related entries. *FOSB* is a member of the Fos gene family (Fos, FosB, FosL1, and FosL2); whose encoded proteins bind to JUN family proteins to form the transcription factor AP-1, which plays an important role in regulating cell proliferation and growth [75]. In studies of ovarian cancer, *FOSB* gene expression serves as a key prognostic indicator in patients undergoing treatment [76]. Zhai et al. analysed ovarian function in small-tailed frigid sheep and reported that melatonin indirectly protects granulosa cells from apoptosis via the FOS pathway [77]. Additionally, the transcription factor AP-1 protein, which involves *FOSB* in its synthesis, plays an important regulatory role in the cervix [78].

The *FGL2* gene encodes a multifunctional protein primarily expressed in macrophages, which may contribute to physiological functions at mucosal sites. This protein is also widely recognized as a as a prognostic biomarker for cancer [79,80]. *FGL2* has been shown to play an important regulatory role in embryo implantation, possibly by affecting endometrial epithelial cell adhesion and thereby facilitating embryo attachment [81,82]. *FGL2* knockout in mice has been reported to cause embryonic developmental insufficiency [83]. In summary, we hypothesise that *FOXC1*, *FOSB*, and *FGL2* are key regulators of litter size in the ovary and uterus of goats. However, further studies are needed to elucidate the specific regulatory mechanisms underlying these hub genes in kidding traits.

In this study, we identified genes and signalling pathways associated with litter size in Jining Grey goats through bioinformatics analyses, including WGCNA and DEG analysis. Additionally, we characterized the biological functions of the turquoise, magenta, green and tan coexpression modules. We also identified 11 hub genes affecting the ovary, 6 hub genes affecting the uterus, and 3 hub genes important for both tissues (*FOXC1*, *FOSB*, and *FGL2*). These findings provide a basis for exploring the potential molecular mechanisms underlying litter size and related traits in goats.

## 5. Conclusions

Among the candidate genes identified in this study, *FOXC1*, *FOSB*, and *FGL2* have emerged as pivotal regulators, significantly influencing both ovarian and uterine functions. By integrating findings from previous studies, we indicate that these genes play critical roles in coordinating reproductive functions across different tissues. Our study reveals candidate genes that contribute to the coordination of reproductive functions between the ovaries and uterus in goats and lays the groundwork for understanding the genetic mechanisms governing reproductive performance in Jining Grey goats.

## Figures and Tables

**Table 1:** Body measurements, weight, and kidding records of Jining Grey Goat.

Group	ID	Age	Body Weight/kg	Body Heigh/cm	Body Length/cm	Chest Circum- Ference/cm	Cannon Bone Circumference/cm	Litter Size		
								First Parity	Second Parity	Third Parity
Polytocous group (P)	P1	3–4	28.5	66	55	78	7	3	5	4
	P2	3–4	32	82	56	83	9	4	5	3
	P3	3–4	30.7	74	53	84	8	4	5	4
	P4	3–4	29.5	67	55	77	8	4	4	3
Monotocous group (M)	M1	3–4	28.6	67	52	75	7.5	1		
	M2	3–4	28.3	73	52	82	8			
	M3	3–4	30.5	68	56	76	8			
	M4	3–4	31.5	73	52	82	8			

## Data Availability

All RNA-seq data generated in this study were submitted to the NCBI SRA database under BioProject No. PRJNA1068677 (RNA-seq).
